# Evaluation of Tropane Alkaloids in Teas and Herbal Infusions: Effect of Brewing Time and Temperature on Atropine and Scopolamine Content

**DOI:** 10.3390/toxins15060362

**Published:** 2023-05-27

**Authors:** Lorena González-Gómez, Sonia Morante-Zarcero, Jorge A. M. Pereira, José S. Câmara, Isabel Sierra

**Affiliations:** 1 ESCET—Escuela Superior de Ciencias Experimentales y Tecnología, Departamento de Tecnología Química y Ambiental, Universidad Rey Juan Carlos, C/Tulipán s/n, Móstoles, 28933 Madrid, Spain; lorena.gonzalez@urjc.es (L.G.-G.); sonia.morante@urjc.es (S.M.-Z.); 2 CQM—Centro de Química da Madeira, Universidade da Madeira, Campus da Penteada, 9020-105 Funchal, Portugal; jorge.pereira@staff.uma.pt (J.A.M.P.); jsc@staff.uma.pt (J.S.C.); 3 Departamento de Química, Faculdade de Ciências Exatas e da Engenharia, Universidade da Madeira, Campus Universitário da Penteada, 9020-105 Funchal, Portugal

**Keywords:** atropine, scopolamine, tropane alkaloids, µSPEed^®^, HPLC–MS/MS, teas/herbal teas, infusions, brewing conditions

## Abstract

Atropine and scopolamine belong to the tropane alkaloid (TA) family of natural toxins. They can contaminate teas and herbal teas and appear in infusions. Therefore, this study focused on analyzing atropine and scopolamine in 33 samples of tea and herbal tea infusions purchased in Spain and Portugal to determine the presence of these compounds in infusions brewed at 97 °C for 5 min. A rapid microextraction technique (µSPEed^®^) followed by high-performance liquid chromatography–tandem mass spectrometry (HPLC–MS/MS) was used to analyze the selected TAs. The results showed that 64% of the analyzed samples were contaminated by one or both toxins. White and green teas were generally more contaminated than black and other herbal teas. Of the 21 contaminated samples, 15 had concentrations above the maximum limit for liquid herbal infusions (0.2 ng/mL) set by Commission Regulation (EU) 2021/1408. In addition, the effects of heating conditions (time and temperature) on atropine and scopolamine standards and naturally contaminated samples of white, green, and black teas were evaluated. The results showed that at the concentrations studied (0.2 and 4 ng/mL), there was no degradation in the standard solutions. Brewing with boiling water (decoction) for 5 and 10 min allowed for higher extraction of TAs from dry tea to infusion water.

## 1. Introduction

Atropine and scopolamine are two natural toxins from the tropane alkaloid (TA) family that are of great concern due to reports of their occurrence in food poisoning events [1,2,3,4,5]. TAs are a large family of alkaloids consisting of more than 200 identified molecules [6,7]. These secondary metabolites with anticholinergic effects occur in different plant families, most notably the Solanaceae family [8,9,10]. However, these toxins also occur in some species of other families, such as Brassicaceae, Convolvulaceae, and Erythroxylaceae. Symptoms of poisoning depend on the amount consumed but are often due to acute toxicity and range from tachycardia, ataxia, and hallucinations to coma or even death due to overdose.

The population is exposed to these natural toxins through food, usually of plant origin, although some studies have reported the transfer of TAs to animal foods, such as honey and milk [3,8]. Cereals and pseudocereals are the most contaminated foods [11,12,13,14,15], followed by teas, herbal teas [16,17,18,19,20], spices [21], honey [22,23], and vegetables [24,25]. Furthermore, over the past few years, the European Rapid Alert System for the Food and Feed (RASFF) portal has reported numerous alerts on different foodstuffs contaminated by TA-producing plants, such as *Atropa belladonna*, *Datura stramonium*, *Mandragora*, *Hyoscyamus niger*, and *Solanum nigrum* [3,26]. Contamination is caused by co-harvested weeds that produce these toxins as they grow between crops, often in more sustainable organic agriculture practices free of pesticides. Popularly, weeds are trawled along with crops during automatic harvesting, resulting in contamination. Awareness of this potential contamination and good manufacturing practices will reduce the risk of mixing toxic and non-toxic plants [3]. However, recently, another route of contamination was proposed [25]. This route involves horizontal transfer through the soil, which has already been proposed for other families of alkaloids, such as pyrrolizidine alkaloids (PAs) [27,28]. Hence, the European Union (EU) has defined maximum limits, generally as the sum of atropine and scopolamine, for certain foods, such as processed and unprocessed cereals and pseudocereals (5–15 ng/g), baby food made from processed cereals and pseudocereals (1 ng/g of atropine and 1 ng/g of scopolamine), dried herbal infusions (25–50 ng/g), and liquid herbal infusions (0.2 ng/g equal to 0.2 ng/mL assuming a density of 1 mg/mL for these beverages) [29]. This regulation entered into force in September 2022, but products legally marketed before this date may remain on the market until their expiration or best-before date.

Despite knowledge about the occurrence of these toxins in food and emerging issues related to TA contamination due to weeds growing among food crops, many questions remain unanswered. For example, studies on the occurrence of TAs in food from associated weeds have mainly focused on cereals (i.e., a seed of stramonium in buckwheat), and there are limited data available for other foods, such as teas and herbal teas [3]. High concentrations of atropine and scopolamine have been found in samples of green tea, black tea, chamomile, peppermint, rooibos, coca leaf tea, verbena, fennel, and nettle, among others [1,16,17,20,21,30,31]. However, most of these studies only involved dry samples. Studies that have focused on the transfer rate of atropine and scopolamine from dried plants to infusions showed transfer rates from 20 to 88% for both toxins [1,21,32]. This wide variation in the transfer rates can be lower due to poor extraction efficiency and eventually because of the heat sensitivity of TAs. Therefore, analysis of liquid infusions is needed to obtain realistic data about TA intake through these beverages. Perhaps atropine and scopolamine should be controlled together with other toxins, such as PAs and other TAs that may appear in this type of sample [4]. Thus, rapidly validated analytical methods with good sensitivity and selectivity are required. Furthermore, to the best of our knowledge, the effects of brewing conditions (time and temperature) on the levels of atropine and scopolamine in tea and herbal tea infusions have not been studied [33].

The aim of this study was to provide data on the presence of atropine and scopolamine in teas and herbal teas (infused water) commercialized in Spain and Portugal between 2021 and 2022 using fast µSPEed^®^ extraction followed by HPLC–MS/MS analysis. Additionally, the influence of brewing conditions (time and temperature) on naturally contaminated white, green, and black teas was also assessed.

## 2. Results and Discussion

### 2.1. Methodology Performance

A methodology that was previously optimized and validated by our group for infusions of chamomile, lemon verbena, lemon balm, green tea, and peppermint was used as a starting point [30]. Parameters, such as linearity, limits of detection and quantification, the matrix effect (ME), selectivity, and recovery, were re-evaluated to determine the performance of the method in the selected samples.

The samples were divided into two groups: tea and herbal infusions. Tea-B-3 and Her-Inf-4 (Table S1) were selected, and matrix-matched calibration curves were created. To achieve this, cold infusions were filtered and doped at increasing concentrations before the µSPEed^®^ procedure. Seven levels were evaluated in a linear range of 0.1–25 ng/mL in the liquid infusion samples. This linear range corresponds to 1.25–313 ng/mL following the methodology, due to the high pre-concentration capacity of the method used (12.5-fold). Linearity was evaluated with the correlation coefficient (R^2^) of each matrix-matched calibration curve, obtained by plotting the peak area of each analyte versus the concentration of each point. The matrix-matched calibration curves for the Tea-B-3 and the Her-Inf-4 infusion samples are listed in Table 1. The results showed good linearity, with R^2^ between 0.995 and 0.998 for both analytes.

In addition, the method detection limit (MDL) and method quantification limit (MQL) were calculated for each sample as 3 and 10 times the standard deviation for the lowest concentration (0.1 ng/mL) in a spiked infusion sample, respectively (Table 1). Low MDLs were found for both analytes (0.02–0.04 for atropine and 0.05 for scopolamine) and MQLs below 0.18 ng/mL for both atropine and scopolamine. These values are lower than the maximum level for liquid herbal infusions (0.2 ng/mL expressed as the sum of atropine and scopolamine) established in Commission Regulation (EU) 2021/1408 [29].

The ME was also estimated by comparing the slopes of the matrix-matched calibration curve with the slope of the solvent calibration curve. The formula used was ME = ((slope matrix-matched calibration/slope solvent calibration) − 1) × 100. The ME was not significant for atropine, as the result did not exceed 20%, as recommended by SANTE guidelines [34]. For scopolamine, a slightly positive ME was found in the case of the Tea-B-3 sample and a slightly negative ME was observed in the case of the Her-Inf-4 sample (Table 1).

Selectivity was assessed for all samples studied. The uncontaminated samples were tested for the absence of a signal at all ion transitions and compared with a sample doped with atropine and scopolamine to verify the total absence of peaks at a retention time (TR) of ±0.1 min. The contaminated samples were also tested for the absence of peaks at a TR of ±0.1 min, and the ion transition ratios were checked in mass resolution units, showing deviations less than 30% (relative abundance).

The recovery percentages for all samples under study were tested at a concentration of 2.5 ng/mL by spiking the infusions with (±)-atropine-D3 and (−)-scopolamine-D3. To perform this test, the area of two spiked infusion samples (pre-extraction spike) was compared with the area of a spiked extract (post-extraction spike). The results demonstrated good application of the methodology optimized and validated by our group for all types of infusion samples [30]. As seen in Table 2, the recovery percentages were 89–109% for atropine and 73–114% for scopolamine. These values are acceptable because they are between 70 and 120%, as specified by SANTE validation guidelines [34].

The methodology used in this work showed good analytical parameters, such as good recovery percentages, as well as other methodologies developed for the determination of atropine and scopolamine in infusion samples [31]. Although the detection limits of this methodology are slightly higher than those proposed by Martinello et al. [31], this methodology complies with the limits required by legislation and presents certain advantages, such as rapid application, obtaining purified extracts ready to be analyzed by HPLC–MS/MS in 12 min. In addition, it generates less residue because this methodology uses small amounts of solvents, and its cartridges are reusable at least 80 times.

### 2.2. Analysis and Quantification of Atropine and Scopolamine in Different Types of Infusions

**Figure 1:**
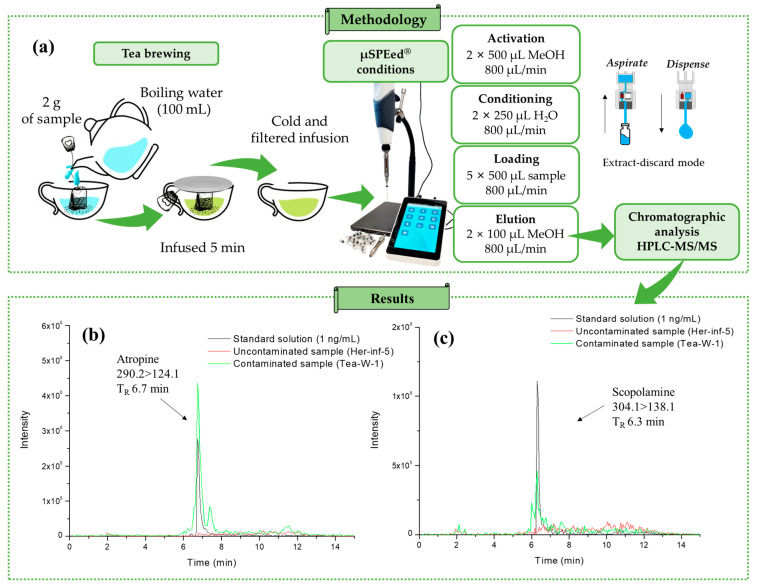
(**a**) Methodology used for sample analysis and extracted ion chromatograms for (**b**) atropine (*m*/*z* 290.1 > 124.0) and (**b**) scopolamine (*m*/*z* 304.1 > 138.0) (**c**) in contaminated Tea-W-1, uncontaminated Herb-Inf-5, and standard solution (1 ng/mL).

A total of 33 samples were studied using the µSPEed^®^ extraction procedure followed by HPLC–MS/MS, as shown in Figure 1a. Matrix-matched calibration curves with internal standards were used for quantification (see Section 4.6) of the Tea-B-3 and Her-Inf-4 samples. To achieve this, 12.5 µL of 500 ng/mL of an internal standard solution containing (±)-atropine-D3 and (−)-scopolamine-D3 was added to 2.5 mL of each filtered infusion before the µSPEed^®^ extraction procedure. The extracts were then analyzed using the HPLC–MS/MS system (Figure 1), and quantification was performed using either the Tea-B-3 calibration curve (for the Tea-B-1–Tea-B-8 samples) or the Her-Inf-4 calibration curve (for the Her-Inf-1–Her-Inf-17 samples). Extracted ion chromatograms (quantification ion) of a sample of white tea (Tea-W-1) infusion contaminated with TAs and an uncontaminated infusion sample of echinacea (Herb-Inf-5), both compared with a standard solution of 1 ng/mL, are shown in Figure 1b,c, respectively.

**Figure 2:**
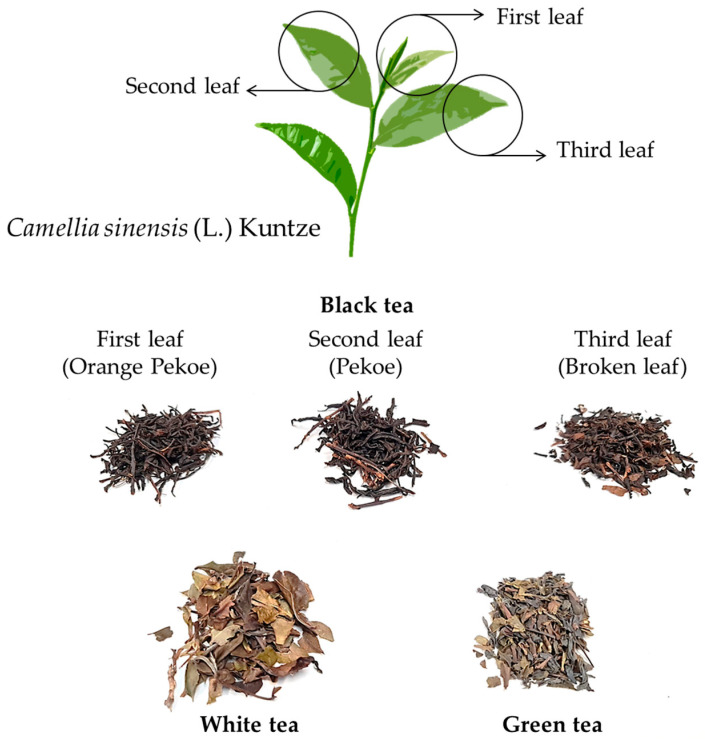
Types of teas from the *Camellia sinensis* (L.) Kuntze plant.

The results of the sample analysis are presented in Table 3. Of the 33 samples tested (Table S1), 21 were contaminated with one or both toxins, corresponding to 64% of the infusion samples studied. In the case of teas, these samples were the most contaminated (Table 3), specifically green and white tea infusions, which generally contain both analytes, whereas black tea infusions were only contaminated with atropine. However, all tea samples exceeded the Commission Regulation (EU) 2021/1408 value (0.2 ng/mL in liquid herbal infusions) [29]. Teas are obtained from the dried leaves of a small tree called *Camellia sinensis* (L.) Kuntze, which belongs to the family Theaceae, D. Don (Figure 2). The different types of teas (white, green, and black) are due to the harvesting and processing steps involved in the tea manufacturing process. Leaves can be collected manually or automatically in all cases [35]. White tea is picked before green and black tea, and buds are picked when they are not open. The processing of this tea is minimal, with only withering and drying of the leaves, leaving the white hairs on the leaves intact to give the appearance of a white color [36]. To produce green tea, the leaves are sterilized with steam after wilting, which prevents their enzymatic oxidation after rolling, since the enzymes (polyphenol oxidase, peroxidase, and catalase) are deactivated [36,37]. There are different types of green tea, one of which is kukicha tea of Japanese origin, also known as twig tea or 3-year tea because the stems and branches used to make it have been on the *Camellia sinensis* (L.) Kuntze plant for at least 3 years, thus losing virtually all theine. In the case of black tea, the leaves are withered, rolled, partially crushed, macerated, and exposed to air, undergoing a slow, natural, and total oxidation process (enzymatic browning). After oxidation, the leaves are dried [35]. There are also different types of black tea, such as first-leaf black tea, also known as Orange Pekoe, which is a whole-leaf tea made up of the upper leaves of the plant, containing a high proportion of buds from the new shoot (Figure 2). Second-leaf black tea, or Pekoe tea, is a lower-quality tea that does not contain budwood, and third-leaf black tea, or broken-leaf tea, is produced from the third leaf of the twig of the tea plant (Figure 2). Sometimes, green and black teas are flavored using fruits and spices, such as ginger, bergamot, and cinnamon (e.g., Pakistani black tea).

Differences in the types of tea appear to influence the content of TAs. Samples of white and green teas, which do not undergo the oxidation process, generally have the highest content of TAs, with levels ranging from 1.24 to 4.94 ng/mL for atropine and around 0.33 ng/mL for scopolamine. However, Tea-G-5 (kukicha green tea) showed the lowest atropine content (≤MQL) compared to the other green teas, and scopolamine contamination was not found. This may be because it is a green tea sample that comes only from the petioles, stems, and twigs of *Camellia sinensis*, allowing for easier differentiation when separating the product from weeds (*Datura stramonium*, *Hyoscyamus niger*, *Solanum nigrum*, etc.) unlike other white or green teas made from leaves. In general, a lower concentration of TAs was found in black teas than in green and white teas, with only atropine being found in black tea samples (between 0.44 and 2.00 ng/mL). Lower TA amounts were observed in Tea-B-5 (0.44 ng/mL) and Tea-B-8 (0.75 ng/mL) from conventional farming.

As shown in Table S1, most of the tea samples were picked from organic crops (pesticides were not used) and probably from tea harvesting machines. When this occurs, the buds of the tea plant are harvested by cutting them together, and weeds can be accidentally cut by the machine. For this reason, leaves that do not comply with quality standards (small tea buds, old tea shoots, etc.) and weeds should be discarded manually. From the results obtained in Table 3, it can be concluded that the cleaning process in the black tea samples is more exhaustive because black tea is a higher-quality product than green and white teas. However, assuming that the B-1, B-2, and B-3 tea samples (organic farming) were manually picked and that cross-contamination with weeds did not occur during their harvesting, the contamination observed could be due to horizontal transfer through the soil because of the existence of TA-producing plants in the vicinity of the tea fields. As can be seen in Table 3, younger parts of the *Camellia sinensis* (L.) Kuntze plant (first and second leaves) have a higher concentration of TAs than older parts (third leaf). These findings are in agreement with those of a study on the contamination source of toxic PAs in tea [38]. In the cited work, the authors reported that the higher concentrations of PAs found in the younger leaves of the tea plant resulted from the transference through the path of PAs producing weed-soil-fresh tea leaves in tea gardens [38].

Finally, it should be noted that the country of origin is not reported on the label of these products because Commission Regulation (EU) 1169/2011 [39] does not mandate declaring the origin of some types of food, such as teas. However, the declaration of the origin would be advisable for tea to help determine the causes of contamination by TAs. A better understanding of the occurrence, invasiveness, and preferred growing conditions of weeds that produce TAs will certainly help farmers identify the sources of tea contamination and take measures to mitigate weed contamination in the fields [3].

In the case of herbal infusion samples (Table 3), only three samples showed atropine, with two of them being below the MQL (0.06 ng/mL), Herb-Inf-4 and Herb-Inf-8. Herb-Inf-7 and Herb-Inf-8 were from fruit-flavored yerba mate (*Ilex paraguarensis*). This is the first time that atropine contamination has been reported in this type of plant. Even though its concentration was lower than the limit established by Commission Regulation (EU) 2021/1408 [29], contamination due to bad harvesting practices is unlikely. Eventually, this may be due to cross-contamination by production lines during the packaging of the product or from soil transference to the plant.

Herb-Inf-9 to Herb-Inf-17 were obtained from herbal mixtures, some flavored and mixed with fruits. Atropine and scopolamine were not found in these samples, except for Herb-Inf-9 and Herb-Inf-11 infusions, which showed atropine contamination below the MQL. The contamination of Herb-Inf-9 may originate from linden, which has been previously reported to have high TA concentrations [30]. In the case of Herb-Inf-11, contamination may come from *Cymbopogon citratus*, although it would also be interesting to investigate the presence of atropine and scopolamine in hibiscus.

Few studies have been performed regarding the presence of TAs in infusion waters. In a previous work, González-Gómez et al. [30] found concentrations of atropine ranging from 0.08 to 3.88 ng/mL and of scopolamine ranging from 0.48 to 1.13 ng/mL in 17 teas and herbal infusions, including chamomile, lemon balm, lemon verbena, green tea, and peppermint. Of these 17 samples, 14 exceeded the limits set by Commission Regulation (EU) 2021/1408 [29]. In the study of Martinello et al. [31], 33 teas and herbal infusions were tested and 4 were found positive for atropine and 5 for scopolamine. The samples included black tea, mint, and a mixture of herbs. Only one sample from this study exceeded the legislated limit of 0.2 ng/mL for liquid infusions, with 0.881 ng/mL for atropine and 1.517 ng/mL for scopolamine. This sample was composed of different herbs, such as liquorice, rhubarb, mallow, and fennel. The data found in this work and the aforementioned studies [30,31] confirm the transfer of these toxins from the dry tea or herbal tea to the brew. Therefore, to assess the realistic exposure of the consumer, these toxins should be determined in infusion waters rather than in dried herbs.

### 2.3. Evaluation of the Effect of Heating/Brewing Conditions on Atropine and Scopolamine

#### 2.3.1. In Standard Solutions

**Figure 3:**
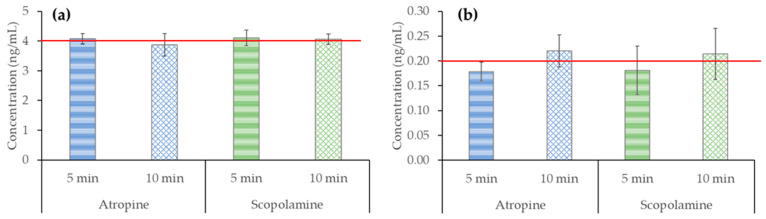
Effect of heating (97 °C maintained for 5 and 10 min) on atropine and scopolamine standard solutions (*n* = 3) at concentrations of (**a**) 4 ng/mL (red line) and (**b**) 0.2 ng/mL (red line).

To evaluate how heating conditions affect the stability of atropine and scopolamine, standard solutions were prepared at two different concentrations, 0.2 and 4 ng/mL. The standards were boiled for 5 and 10 min, simulating the more drastic brewing technique by decoction (see Section 4.3.1). The peak areas obtained after HPLC–MS/MS analysis were interpolated into solvent calibration curves. The results obtained are shown in Figure 3. As can be seen, no degradation was observed in standard solutions at any of the concentrations or times studied. These results demonstrate that atropine and scopolamine are quite heat-stable under the studied conditions and that the brewing techniques usually used to prepare infusions (97 °C and allowed to cool for 5 min) or decoctions (boiling for 5 and 10 min) do not affect the presence of these toxins in the brew. This is an important point because under real tea-making conditions, the TAs found in the weed impurities of tea samples would be less exposed to heat in the plant matrix than when they are added as standard solutions, so they are more likely heat-stable.

A previous study by Martín-Sáez et al. [32] that simulated tea-making conditions in vials spiked with standard solutions (100 °C, left to cool for 5 min) showed degradation of atropine and scopolamine by up to 32%. However, this study was carried out at a high concentration, specifically 5000 ng/mL, which is 4 orders of magnitude higher than the maximum amount allowed for liquid herbal infusions (0.2 ng/mL).

#### 2.3.2. In Contaminated Green, White, and Black Tea Samples

**Figure 4:**
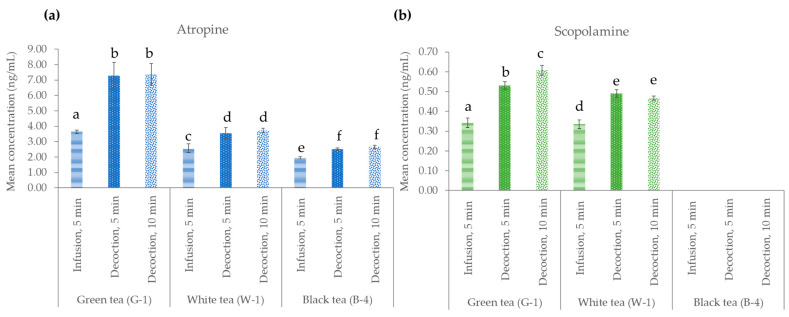
Effect of brewing conditions on tea preparation with green (G-1), white (W-1), and black (B-4) tea samples naturally contaminated with (**a**) atropine and (**b**) scopolamine: infusion with water at 97 °C with cooling (5 min) or decoction at 97 °C for 5 and 10 min. ANOVA was performed with Duncan’s test, showing the same letters in the figure when there were no statistically significant differences, and different letters indicate that there were such differences (*p* ≤ 0.05).

In this section, the effect of temperature on the preparation of naturally contaminated green (G-1), white (W-1), and black (B-4) teas was evaluated. For this purpose, two tea brewing times, 5 and 10 min, while maintaining the temperature at 97 °C were studied (see Section 4.3.2 and Figure 1a). This study was compared with the procedure described in the international standard ISO 3103, which is described in Section 4.2, and applied to the analysis of all samples in this work. The results are shown in Figure 4. ANOVA was applied with Duncan’s test to evaluate statistically significant differences between the conditions studied. As can be seen, the TA concentration increased in the decoction procedure compared to the infusion conditions, and in general, the increase in the decoction time (from 5 to 10 min) did not produce a significant increase in the TA content. Maintaining the temperature at 97 °C for 10 min showed a concentration of 7.38 ± 0.70 ng/mL for atropine and 0.61 ± 0.03 ng/mL for scopolamine in the brew obtained from the G-1 sample. These results showed that decoction conditions allow a higher transfer rate of atropine and scopolamine from the (weed) dry plant to the infusion.

Considering the results obtained in this work, it is advisable to avoid the preparation of infusions by maintaining the boiling temperature, as this temperature does not allow degradation of the toxins studied but rather a greater extraction of them that would increase the intake of these toxins. To contribute to a better understanding of how TAs are transferred to infusion drinks, it would be advisable to study the transfer of TAs from dry infusions doped with TA-producing plants to infusion water. Depending on the type of TA-producing plant and the influence of the matrix of the contaminated sample, there may be different transfer percentages, as already demonstrated in herbal infusions contaminated with PA-producing plants [40].

## 3. Conclusions

This study presented data on the occurrence of atropine and scopolamine in infusion samples using the fast and sustainable microextraction technique µSPEed^®^. The results indicated that 64% of the samples tested had atropine or scopolamine contamination in the brewing water after tea processing, confirming that not only does contamination occur in the dry sample but also that these compounds are transferred to the infusion. This fact highlights the need for exhaustive control of this type of food. Additionally, this study revealed the resistance of the referred TAs in standard solutions at 97 °C and short times of 5 and 10 min, respectively, and demonstrated that the infusion preparation significantly influences their extraction.

Based on the results obtained, it is recommended to determine the TA content of the infusions rather than the dry herbs to limit the overestimation of the real intake of TAs through the ingestion of these beverages. Brewing conditions, such as steeping time and temperature, influence the transfer rates for TAs, but other factors may also play a role, such as the tea or herbal tea type, the herb-to-water ratio, the pH of the infusion, heat dissipation of the vessel, the particle size of the dry herbs, or the TA concentration, among others. Therefore, to assess realistic consumer exposure, it is preferable to prepare brews according to the manufacturer’s instructions.

## 4. Materials and Methods

### 4.1. Chemicals, Reagents, and Standard Solutions

Organic solvents, such as acetonitrile (ACN) and methanol (MeOH) LC-MS grade, were purchased from Scharlab (Barcelona, Spain), while formic acid (≥99%) Optima™ LC-MS grade was obtained from Fisher Chemical (Madrid, Spain). Ultrapure deionized water (18.2 MΩ cm quality) was obtained using the Millipore Milli-Q-System (Billerica, MA, USA) and was used for mobile phases, aqueous solutions, and the preparation of tea and herbal infusions. (±)-Atropine-D3 (1 mg, MW 292.39 g/mol) was acquired from Análisis Vínicos (Tomelloso, Spain), while (−)-scopolamine-D3 hydrochloride solution (100 µg/mL in ACN:Water (9:1), MW 342.83 g/mol; CAS 1202357-61-6), scopolamine hydrobromide (≥98%, CAS 6533-68-2), and atropine (≥99%, MW 289.37 g/mol CAS 51-55-8) were obtained from Sigma-Aldrich (St. Louis, MO, USA). Atropine and scopolamine individual stock standard solutions were prepared in MeOH at a concentration of 1000 µg/mL. Internal standards, such as (±)-atropine-D3, were prepared in 10 mL of MeOH (100 µg/mL). In contrast, (−)-scopolamine-D3 hydrochloride solution was diluted in 10 mL of MeOH (10 µg/mL). Working solutions were prepared by diluting in MeOH and stored in the dark at −20 °C until use. A solvent calibration curve (0.1–25 ng/mL) was prepared by diluting with MeOH. Nylon syringe filters (0.45 µm, 13 mm) used to filter the samples before the µSPEed^®^ stage were obtained from Mervilab (Madrid, Spain). The digiVOL^®^ Digital Syringe and cross-linked polystyrene divinylbenzene (PS/DVB, 3 μm/300 Å) cartridges were purchased from EPREP (Mulgrave, VI, Australia).

### 4.2. Sample and Infusion Preparations

A total of 33 samples of teas and herbal teas purchased from different local markets in Spain and Portugal were evaluated. Table S1 provides detailed information about the type of sample, scientific name, ingredients, country of acquisition, and type of farming. The infusion samples were prepared according to the international standard ISO 3103 protocol [41]. To do this, 2 g of each sample was weighed into disposable tea bags, and ultrapure deionized water was heated in a kettle until 97 °C. The tea bags were then placed in a cup together with 100 mL of boiling water. The cup was capped and left to cool for 5 min to ensure proper infusion of the plant. The sample was then cooled in a refrigerator and filtered with a nylon filter before using the µSPEed^®^ extraction procedure (Figure 1a). Each sample was prepared in triplicate following the procedure described earlier.

### 4.3. Studies to Evaluate the Effect of Heating/Brewing Conditions on Atropine and Scopolamine

#### 4.3.1. In Standard Solutions

The effect of heating conditions on standard solutions was evaluated. For this purpose, 100 mL of water was heated to 97 °C. Once the desired temperature was reached, the water was doped with the target analytes at two concentrations, 0.2 ng/mL and 4 ng/mL, and boiling was maintained for 5 or 10 min. The concentrations were selected according to the maximum contents found in the samples analyzed in this work and the maximum levels of atropine and scopolamine for liquid plant infusions (0.2 ng/mL) specified in Commission Regulation (EU) 2021/1408 [29]. After the required time had elapsed (5 or 10 min), the water was allowed to cool to room temperature, and the volume was measured and made up to 100 mL. Each test was performed in triplicate *(n* = 3). Finally, the µSPEed^®^ protocol described in Section 4.4 was performed before HPLC–MS/MS analysis.

#### 4.3.2. In Contaminated Green, White, and Black Tea Samples

To evaluate the effect of brewing conditions on the content of atropine and scopolamine in the beverages, previously analyzed tea samples contaminated with both TAs (G-1, W-1 and B-4) were selected. Two domestic brewing techniques were compared: decoction and infusion. To make tea decoction, 100 mL of ultrapure deionized water was heated to 97 °C, and then, a disposable tea bag containing 2 g of the G-1, W-1, or B-4 tea sample was introduced and left to simmer for 5 or 10 min (during this time, the temperature was maintained at 97 °C). After this time, the disposable tea bag with the sample was removed, and the infusion was allowed to cool to room temperature. The volume of the infusion was measured and made up to 100 mL. The sample was then filtered, 2.5 mL of the sample was taken, and the protocol described in Section 4.4 was applied before HPLC–MS/MS analysis. The results of this brewing technique were compared with the infusion prepared following the protocol described in Section 4.2. These tests were performed in triplicate (*n* = 3). ANOVA with Duncan’s test was then performed on the results obtained using SPSS statistical software.

### 4.4. µSPEed^®^ Extraction Procedure

The infusion samples were purified and preconcentrated following the protocol previously established [30]. The optimized and validated µSPEed^®^ procedure was applied using a polymeric-based cartridge, specifically cross-linked polystyrene divinylbenzene (PS/DVB, 3 μm/300 Å). The protocol involved a conditioned step with water (2 × 250 µL), a loading step with five 500 µL aliquots of the filtered infusion sample (5 × 500 µL) in extract-discard mode, and an elution step with two 100 µL aliquots of MeOH (2 × 100 µL). The flow rate of 800 µL/min was constant throughout the µSPEed^®^ procedure. The purified extract was directly injected into the HPLC–MS/MS equipment. To maintain and reuse the cartridge, it was washed twice with 500 µL MeOH (2 × 500 µL) at the end of each extraction. The cartridge was activated in the same way between days, and each cartridge was reused approximately 80 times (Figure 1a).

### 4.5. HPLC–MS/MS Conditions

Chromatographic analysis was performed using Varian 1200/1200 LC (Varian Ibérica, Spain) with a ProStar 410 autosampler (100 µL loop), two ProStar 210/215 solvent delivery modules, and a thermostatized space for the column. Separation was carried out following our previous work [30]. A C18 Kromaphase 100 column (150 mm × 2.0 mm, 3.5 μm particle size; Scharlab, Barcelona, Spain) with a C18 Kromaphase guard column (10 mm × 4.0 mm, 5 μm particle size; Scharlab, Barcelona, Spain) was used at 30 °C. The injection volume was 10 µL (partial injection), and the flow rate was set at 0.25 mL/min. The mobile phases consisted of solvent A (Milli-Q water) and solvent B (ACN), both containing 0.1% formic acid. The separation was in gradient elution started at 90% A and then decreased linearly to 30% in 10 min and returned to 90% in 1 min, holding these conditions for 4 min. The total run time of the method was 15 min.

For detection, a triple quadrupole (1200 L TQ) mass spectrometer detector with an electrospray ionization (ESI) ion source operating in positive mode with the multiple reaction monitoring (MRM) mode (mass peak width Q1 2.5, mass peak width Q3 2.5, scan width in MRM 0.70) was used. The data acquisition system was MS Workstation version 6.8. N_2_ was used as a drying gas and as a nebulizer gas. Argon was used as a collision gas. The conditions were as follows: N_2_ drying gas (350 °C, 22 psi), nebulizer gas pressure (58 psi), capillary voltage (5000 V), and shield (600 V). Argon was set at 1.90 mTorr and the detector voltage at 1535 V. The TAs were monitored at a cone voltage of 70 V and a dwell time of 0.25 s following the transitions shown in Table S2. Quantification was performed using the next product ions: 124.1 *m*/*z* for atropine, 127.2 *m*/*z* for (±)-atropine-D3, 156.0 *m*/*z* for scopolamine, and 159.0 *m*/*z* for (−)-scopolamine-D3. The retention time was 6.3 min for scopolamine and (−)-scopolamine-D3 and 6.7 min for atropine and (±)-atropine-D3.

### 4.6. Quantification of Atropine and Scopolamine

To quantify the analytes, matrix-matched calibration curves were prepared using an internal standard. Two calibration curves, each with seven points at concentrations ranging from 0.1 to 25 ng/mL (in the infusion sample), were prepared using Tea-B-3 and Her-Inf-4. To prepare these curves, 2.5 mL of the filtered sample was taken and spiked with increasing and appropriate concentrations of atropine and scopolamine. Next, a 12.5 µL aliquot of a 500 ng/mL solution containing (±)-atropine-D3 and (−)-scopolamine-D3 was added. The protocol described in Section 4.4 was then followed, and the resulting aliquots were injected into the HPLC–MS/MS equipment following the conditions described in Section 4.5.

Calibration curves were constructed using the ratio of the analyte peak area for the 124.1 *m*/*z* ion for atropine and the 156.0 *m*/*z* ion for scopolamine to the internal standard peak area for the 127.2 *m*/*z* ion for (±)-atropine-D3 and the 159.0 *m*/*z* ion for (−)-scopolamine-D3 versus the analyte concentration. All samples were spiked with the same amount of internal standard (2.5 ng/mL) used in the calibration curves before analysis. After analysis, the areas of the identified atropine or scopolamine peaks found in the samples were divided by the area of the internal standard and interpolated onto the internal standard calibration curves. Each sample was analyzed in triplicate, and the mean and standard deviation of the obtained concentrations were calculated. Concentrations were expressed in ng/mL. The density of Tea-B-3 was 1.0017 g/mL and of Her-Inf-4 was 1.0020 g/mL.

## Figures and Tables

**Table 1:** Analytical performance of the µSPEed^®^ procedure for atropine (At) and scopolamine (Sc) determination in Tea-B-3 and Her-Inf-4 samples.

Calibration (Sample)	Linearity for At 1.25–313 ng/mL (R^2^) ^a^	Linearity for Sc 1.25–313 ng/mL (R^2^) ^a^	MDL ^b^ (ng/mL)		MQL ^c^ (ng/mL)		ME ^d^ (%)	
			At	Sc	At	Sc	At	Sc
Matrix-matched calibration (Tea-B-3)	2.2 × 10^6^x + 4.1 × 10^6^ (0.995)	8.6 × 10^5^x − 1.1 × 10^6^ (0.996)	0.04	0.05	0.14	0.18	10	23
Matrix-matched calibration (Her-Inf-4)	1.7 × 10^6^x + 2.9 × 10^6^ (0.998)	4.7 × 10^5^x + 2.0 × 10^6^ (0.996)	0.02	0.05	0.06	0.18	−15	−33

^a^ The linear range corresponds to 0.1–25 ng/mL in the infusion sample according to the validated analytical methodology. ^b^ MDL: method detection limit. ^c^ MQL: method quantification limit. ^d^ Matrix effect (ME) = ((slope matrix-matched calibration/slope solvent calibration) − 1) × 100. Solvent calibration: *y* = 2.0 × 10^6^x + 2.1 × 10^6^ (R^2^ 1.000) for atropine and *y* = 7.0 × 10^5^x − 1.6 × 10^6^ (R^2^ 0.999) for scopolamine.

**Table 2:** Recovery percentages (*n* = 3) evaluated for all infusion samples studied at a concentration of 2.5 ng/mL.

Infusion Code	(±)-Atropine-D3 (Recovery % ± SD)	(−)-Scopolamine-D3 (Recovery % ± SD)
Tea-W-1	101 ± 3	98 ± 8
Tea-W-2	103 ± 4	101 ± 5
Tea-W-3	89 ± 9	81 ± 9
Tea-G-1	102 ± 1	95 ± 2
Tea-G-2	102 ± 1	105 ± 3
Tea-G-3	97 ± 6	103 ± 1
Tea-G-4	106 ± 5	102 ± 2
Tea-G-5	97 ± 1	102 ± 2
Tea-B-1	97 ± 1	100 ± 1
Tea-B-2	104 ± 1	106 ± 4
Tea-B-3	107 ± 1	103 ± 5
Tea-B-4	101 ± 3	99 ± 7
Tea-B-5	96 ± 5	89 ± 3
Tea-B-6	102 ± 3	94 ± 9
Tea-B-7	99 ± 1	90 ± 1
Tea-B-8	105 ± 4	92 ± 2
Her-Inf-1	103 ± 8	73 ± 3
Her-Inf-2	102 ± 5	99 ± 3
Her-Inf-3	97 ± 4	104 ± 2
Her-Inf-4	98 ± 4	86 ± 9
Her-Inf-5	106 ± 1	97 ± 4
Her-Inf-6	104 ± 9	103 ± 11
Herb-Inf-7	108 ± 8	114 ± 2
Herb-Inf-8	106 ± 5	102 ± 4
Herb-Inf-9	103 ± 1	73 ± 7
Herb-Inf-10	101 ± 1	87 ± 1
Herb-Inf-11	102 ± 1	98 ± 1
Herb-Inf-12	103 ± 6	106 ± 4
Herb-Inf-13	98 ± 1	85 ± 3
Herb-Inf-14	105 ± 1	92 ± 6
Herb-Inf-15	109 ± 9	96 ± 1
Herb-Inf-16	92 ± 8	93 ± 8
Herb-Inf-17	93 ± 3	96 ± 3

**Table 3:** Contents of atropine and scopolamine in the tea and herbal tea infusions analyzed.

Infusion Code	Sample Description (Code)	Atropine (ng/mL ± SD)	Scopolamine (ng/mL ± SD)
Tea-W-1 ^a^	White tea (W-1)	2.57 ± 0.28	0.33 ± 0.02
Tea-W-2 ^a^	White tea (W-2)	1.24 ± 0.14	0.33 ± 0.04
Tea-W-3 ^a^	White tea (W-3)	2.86 ± 0.12	ND
Tea-G-1 ^a^	Green tea (G-1)	3.65 ± 0.10	0.34 ± 0.02
Tea-G-2 ^a^	Green tea (G-2)	4.65 ± 0.57	ND
Tea-G-3 ^a^	Green tea (G-3)	4.94 ± 0.74	≤MQL
Tea-G-4 ^a^	Green tea (G-4)	4.15 ± 0.24	ND
Tea-G-5 ^a^	Kukicha green tea (G-5)	≤MQL	ND
Tea-B-1 ^a^	Black tea (B-1)	1.81 ± 0.14	ND
Tea-B-2 ^a^	Black tea (B-2)	1.65 ± 0.07	ND
Tea-B-3 ^a^	Black tea (B-3)	1.30 ± 0.16	ND
Tea-B-4 ^a^	Black tea with bergamot (B-4)	1.95 ± 0.08	ND
Tea-B-5 ^a^	Black tea (B-5)	2.00 ± 0.16	ND
Tea-B-6 ^a^	Black tea (B-6)	0.44 ± 0.05	ND
Tea-B-7 ^a^	Pakistani black tea (B-7)	1.74 ± 0.08	ND
Tea-B-8 ^a^	Black tea (B-8)	0.75 ± 0.03	ND
Her-Inf-1 ^b^	Pink lapacho bark tea (Her-1)	ND	ND
Her-Inf-2 ^b^	Lemon grass tea (Her-2)	ND	ND
Her-Inf-3 ^b^	Rosemary (Her-3)	ND	ND
Her-Inf-4 ^b^	Valerian (Her-4)	≤MQL	ND
Her-Inf-5 ^b^	Echinacea (Her-5)	ND	ND
Her-Inf-6 ^b^	Star anise (Her-6)	ND	ND
Herb-Inf-7 ^b^	Flavored yerba mate (Her-7)	0.14 ± 0.01	ND
Herb-Inf-8 ^b^	Flavored yerba mate (Her-8)	≤MQL	ND
Herb-Inf-9 ^b^	Mixed herbal tea (Her-9)	<MQL	ND
Herb-Inf-10 ^b^	Mixed herbal tea (Her-10)	ND	ND
Herb-Inf-11 ^b^	Mixed herbal tea (Her-11)	<MQL	ND
Herb-Inf-12 ^b^	Mixed herbal tea (Her-12)	ND	ND
Herb-Inf-13 ^b^	Mixed herbal tea (Her-13)	ND	ND
Herb-Inf-14 ^b^	Mixed herbal tea (Her-14)	ND	ND
Herb-Inf-15 ^b^	Mixed herbal tea (Her-15)	ND	ND
Herb-Inf-16 ^b^	Mixed herbal tea (Her-16)	ND	ND
Herb-Inf-17 ^b^	Mixed herbal tea (Her-17)	ND	ND

^a^ Internal standard calibration curves using Tea-B-3 as the matrix for quantification (linear range: 0.1–25 ng/mL in the infusion sample): atropine (y = 0.022x + 0.009, R^2^ 0.999) and scopolamine (y = 0.028x − 0.045, R^2^ 0.994). ^b^ Internal standard calibration curves using Her-Inf-4 as the matrix for quantification (linear range: 0.1–25 ng/mL in the infusion sample): atropine (y = 0.025x + 0.075, R^2^ 0.998) and scopolamine (y = 0.028x − 0.092, R^2^ 0.999). ≤MQL: below or equal to the limit of quantification of the method (0.14 ng/mL for atropine and 0.18 ng/mL for scopolamine); ND: not detected.

## Data Availability

Not applicable.

## Supplementary Materials

The following supporting information can be downloaded at https://www.mdpi.com/article/10.3390/toxins15060362/s1: Table S1: Description of samples analyzed; Table S2: Parameters of mass spectrometry analysis in positive ionization mode.
