# Supplementary material for: Evaluation of Tropane Alkaloids in Teas and Herbal Infusions: Effect of Brewing Time and Temperature on Atropine and Scopolamine Content
Source: Toxins (Basel). 2023 May 27;15(6):362. doi: 10.3390/toxins15060362 (PMC10301465; doi:10.3390/toxins15060362)
Supplement: Supplementary file 1 [file toxins-15-00362-s001.zip › toxins-2383549-supplementary.pdf]

Supplementary Materials

Evaluation of Tropane Alkaloids in Teas and Herbal Infusions: Effect of Brewing Time and Temperature on Atropine and Scopolamine Content

Lorena González-Gómez[1], Sonia Morante-Zarcero[1], Jorge A.M. Pereira[2], José S. Câmara[2,3] and Isabel Sierra[1,*]

[1]ESCET- Escuela Superior de Ciencias Experimentales y Tecnología, Departamento de Tecnología Química y Ambiental, Universidad Rey Juan Carlos, C/Tulipán s/n, 28933 Móstoles, Madrid,Spain

[2]CQM – Centro de Química da Madeira, Universidade da Madeira, Campus da Penteada, 9020-105 Funchal, Portugal

[3]Departamento de Química, Faculdade de Ciências Exatas e da Engenharia, Universidade da Madeira, Campus Universitário da Penteada, 9020-105 Funchal, Portugal

*Correspondence: isabel.sierra@urjc.es; Tel.: +34-91-488-7018; Fax: +34-91-488-8143

Table S1. Description of samples analysed

Infusion code	Sample description (code)	Scientific name or ingredients	Purchased	Format	Type of farming
Tea-W-1	White tea (W-1)	*Camellia sinensis*	S	Leaves	Conventional
Tea-W-2	White tea (W-2)	*Camellia sinensis*	P	Leaves	Organic
Tea-W-3	White tea (W-3)	*Camellia sinensis*	S	Ground	Organic
Tea-G-1	Green tea (G-1)	*Camellia sinensis* (top three leaves)	P	Leaves	Organic
Tea-G-2	Green tea (G-2)	*Camellia sinensis* (95 %), natural coconut aroma (5%)	S	Leaves	Conventional
Tea-G-3	Green tea (G-3)	*Camellia sinensis*	P	Leaves	Organic
Tea-G-4	Green tea (G-4)	*Camellia sinensis*	S	Ground	Conventional
Tea-G-5	Kukicha green tea (G-5)	*Camellia sinensis* (only petioles, stems and twigs)	S	Ground	Organic
Tea-B-1	Black tea (B-1)	*Camellia sinensis* (first leaf)	P	Leaves	Organic
Tea-B-2	Black tea (B-2)	*Camellia sinensis* (second leaf)	P	Leaves	Organic
Tea-B-3	Black tea (B-3)	*Camellia sinensis* (third leaf)	P	Leaves	Organic
Tea-B-4	Black tea with bergamot (B-4)	*Camellia sinensis* (60 %), bergamot aroma black tea (40 %)	P	Leaves	Organic
Tea-B-5	Black tea (B-5)	*Camellia sinensis*, centaury and aroma	S	Leaves	Organic
Tea-B-6	Black tea (B-6)	*Camellia sinensis*	S	Leaves	Conventional
Tea-B-7	Pakistani black tea (B-7)	*Camellia sinensis* (60 %), cassia cinnamon (15 %), ginger (14 %) and flavours	S	Leaves/Fruits	Conventional
Tea-B-8	Black tea (B-8)	*Camellia sinensis*	S	Leaves	Conventional
Her-Inf-1	Pink lapacho bark tea (Her-1)	*Handroanthus impetiginosus*	P	Bark	Conventional
Her-Inf-2	Lemon grass tea (Her-2)	*Cymbopogon citratus*	P	Leaves	Conventional
Her-Inf-3	Rosemary (Her-3)	*Rosmarinus officinalis L.*	S	Leaves	Organic
Her-Inf-4	Valerian (Her-4)	*Valeriana officinalis*	S	Leaves	Organic
Her-Inf-5	Echinacea (Her-5)	*Echinacea purpurea (L.) moench*	S	Leaves/flowers	Organic
Her-Inf-6	Star anise (Her-6)	*Pimpinella anisum*	S	Fruit	Conventional
Herb-Inf-7	Flavoured yerba mate (Her-7)	Yerba mate (*Ilex paraguarensis,* 89 %), flavouring, mandarine peel (1 %)	P	Leaves	Conventional

Table S1. Cont.

Infusion code	Sample description (code)	Scientific name or ingredients	Purchased	Format	Type of farming
Herb-Inf-8	Flavoured yerba mate (Her-8)	Yerba mate (*Ilex paraguarensis,* 84 %), flavouring, liquorice root (5 %), grapefruit peel (2 %), fig (2%)	P	Leaves	Conventional
Herb-Inf-9	Mixed herbal tea (Her-9)	Apple pulp (*Malus domestica,* 42.6%), cinnamon (*Cinnamomum spp*, 11%), apple (*Malus domestica,* 10%), rose hips (*Rosa canina*), chicory root (*Cichorium intybus*), Chinese mulberry leaves (*Rubus suavissimus S.Lee*), hibiscus (*Hibiscus sabdariffa*), apple aroma, licorice root (*Glycyrrhiza glabra*), linden (tilia argentea/tilia tomentosa), apple/cinnamon aroma (sulfites), acid (malic acid)	P	Leaves/ Dried fruits	Conventional
Herb-Inf-10	Mixed herbal tea (Her-10)	Rosehip, apple (*Malus domestica,* 35%), hibiscus (20%), ash leaves, meadowsweet, red fruits (2%, blackberries, rasperries, strawberries), blackberry and vanilla flavour.	P	Leaves/ Dried fruits	Conventional
Herb-Inf-11	Mixed herbal tea (Her-11)	Lemongrass (*Cymbopogon citratus,* 33%), rose hips (*Rosa canina,* 18%), orange leafs, orange peel (8%), hibiscus (5%), elder, orange flavour.	P	Leaves/ Dried fruits	Conventional
Herb-Inf-12	Mixed herbal tea (Her-12)	Forest fruits: apple, elderberries, rose hips, raspberry aroma, blackberries and strawberries in variable portions. Hibiscus and aromas.	S	Leaves/ Dried fruits	Conventional
Herb-Inf-13	Mixed herbal tea (Her-13)	Apple, hibiscus, orange, lemon, lemongrass, flavourings, acid: tartaric acid, orange granules (orange juice concentrate and orange pieces), lemon granules (glucose syrup, lemon juice concentrate, sweetened apricot, modified starch, thickener: sodium alginate and aroma)	S	Leaves/ Dried fruits	Conventional

Table S1. Cont.

Infusion code	Sample description (code)	Scientific name or ingredients	Purchased	Format	Type of farming
Herb-Inf-14	Mixed herbal tea (Her-14)	Lemon balm, peppermint, fennel, mallow, pennyroyal balm-of-gilead, lemon verbena, lemon grass	P	Leaves	Conventional
Herb-Inf-15	Mixed herbal tea (Her-15)	Rooibos (*Aspalathus linearis,* 70 %), basil (30%)	S	Leaves	Conventional
Herb-Inf-16	Mixed herbal tea (Her-16)	Rooibos (*Aspalathus linearis*, 89.5%), natural flavours (3%), orange peel (2%), lemon peel (2%), sunflower (1%), rose (1%), orange blossom (1%) and cornflower (0.5%).	S	Leaves	Conventional
Herb-Inf-17	Mixed herbal tea (Her-17)	Rooibos, pineapple (pineapple, sugar, acid (citric acid)), coconut, flavouring and sunflower	S	Leaves	Conventional

P: Portugal; S: Spain

Table S2. Parameters of mass spectrometry analysis in positive ionization mode.

Analyte	Precursor Ions (Q_1, m/z, $[M+H]^+$)	Capillary (V)	MS^2 Product Ions (Q_3, m/z)	CE (V)	Dwell Time (s)
Atropine	290.2	70	90.9	34	0.25
			93.0	29	0.25
			124.1 *	20.5	0.25
(±)-Atropine-D3	293.4	70	92.9	25	0.25
			102.7	42	0.25
			121.3	28	0.25
			127.2 *	21	0.25
Scopolamine	304.1	70	121.0	16	0.25
			138.1 *	12	0.25
			156.0	9.5	0.25
(-)-Scopolamine-D3	307.4	70	121.1	24.5	0.25
			141.2 *	24.5	0.25
			159.0	9	0.25
